# Spatiotemporal dynamics and environmental trends of reported human leptospirosis in Sri Lanka, 2007–2024

**DOI:** 10.3389/fpubh.2026.1814638

**Published:** 2026-05-18

**Authors:** Ian A. McMillan, Prashant Dahal, Md Samun Sarker, Thi Hai Au La, Jourdan K. P. McMillan, Sandra P. Chang, Siriwardana Rampalage Sarathchandra, Chandika Gamage, Michael H. Norris

**Affiliations:** 1 Daniel K. Inouye Center for Microbial Oceanography: Research and Education, School of Ocean and Earth Science and Technology, University of Hawai’i at Mānoa, Honolulu, HI, United States; 2 Pathogen Analysis and Translational Health Group, School of Life Sciences, University of Hawai’i at Mānoa, Honolulu, HI, United States; 3 Department of Tropical Medicine, Medical Microbiology & Pharmacology, John A. Burns School of Medicine, University of Hawai’i at Mānoa, Honolulu, HI, United States; 4 Rice Research and Development Institute, Batalagoda, Sri Lanka; 5 Department of Microbiology, Faculty of Medicine, University of Peradeniya, Peradeniya, Sri Lanka

**Keywords:** *Leptospira*, leptospirosis, *lipL32*, neglected tropical disease, Sri Lanka, one health, zoonotic disease

## Abstract

Leptospirosis is a globally significant zoonoses that contributes to 1.03 million infections and 58,900 deaths annually. Animal reservoirs carry pathogenic *Leptospira* in the kidneys and contaminate the environment via urine. Contact of mucous membranes or abrasions with pathogenic *Leptospira* commonly leads to infection in humans. Leptospirosis has various presentations, ranging from an acute self-limiting infection to a severe infection with multi-organ involvement requiring hospitalization. In 2008, leptospirosis cases in Sri Lanka spiked, marking a potential emergence event. In the current work, cases of reported leptospirosis were analyzed from 2007 to 2024 identifying an overall increase in leptospirosis over the study period and annual seasonality. Increased precipitation seems to be driving the seasonal spikes in leptospirosis case rates across Sri Lanka. District level case rates over the 18-year time frame increased in the southern districts and indicated a dissemination to northern districts. A low-low spatial cluster, signifying an area of lower disease, was identified in the northern districts from 2007 to 2012, but was not observed from 2013 to 2024. A high-high spatial cluster, indicating an area of increased incidence, was identified from 2013 to 2024. District level precipitation and case rates were correlated over the 18-year study period, suggesting that rainfall is a driver of disease in Sri Lanka. While this correlation was identified to be significant, the correlation weakens over time when analyzed per annum with 2011 and 2024 showing no link. No correlation was identified in 2011, however, analysis of precipitation trends and case rates over the year show that major flooding events drove anomalous increases in leptospirosis case rates throughout the country. In 2024, a record year in reported leptospirosis, there was also no correlation over the entire year but the seasonality of disease still aligns with the overall trend. Investigation of renal carriage in five potential animal reservoirs identified that all carried the *lipL32* gene of pathogenic *Leptospira* species. Together, this work identified temporal and spatial trends of human leptospirosis in Sri Lanka, identifying a link with precipitation as a potential driver of disease, and further characterized localized renal carriage of animal reservoirs linked to environmental contamination.

## Introduction

Leptospirosis is a tropical zoonotic disease that causes significant morbidity throughout the world. Infection with a pathogenic strain of *Leptospira* can lead to leptospirosis, culminating in diverse clinical presentations ranging from subclinical infection with undifferentiated fever to jaundice with renal failure and pulmonary hemorrhage (1). Severe manifestations of leptospirosis, including multiple organ failure, acute kidney injury, and pulmonary hemorrhage syndrome, can lead to mortality rates of 50% (2–9). Less severe forms can be misdiagnosed as other tropical infections such as malaria or dengue fever, obscuring the actual burden of leptospirosis globally (10–14). Currently, the global burden of leptospirosis is estimated at 1.03 million infections each year, with a mortality rate of 5.72% (15). Sequelae and death associated with leptospirosis contribute an estimated 2.9 million disability adjusted life years (DALYs), a burden similar to schistosomiasis, lymphatic filariasis, and leishmaniasis (16). The global burden of leptospirosis is also estimated to be 70% of cholera and twice that of rabies (16). Despite these similarities, leptospirosis is underrecognized as a neglected tropical disease (17). A disproportionate amount of leptospirosis burden occurs in resource-poor countries, although significant rates are still reported in developed countries (15, 16).

Transmission and spread of leptospirosis is complex and involves many variables. Pathogenic *Leptospira* circulate within many different animal reservoirs through persistent carriage in the kidneys followed by shedding into the environment through urine (18). While rodents, such as *Rattus* spp., are considered a primary reservoir, many other wild and domestic mammals can also contribute to the ecology of pathogenic *Leptospira* (19–28). After contaminated urine enters the environment, pathogenic *Leptospira* can survive for weeks to months in water and soil (29, 30). Transmission to humans occurs through contact of mucous membranes or damaged skin with contaminated water, soil, or animal secretions during activities associated with occupation, recreation and extreme weather events (31–39). The presence of animal reservoirs, landscape, and climate contribute to leptospirosis disease transmission and dynamics. For example, in urban areas lacking significant sanitation infrastructure and rodent control, rat populations can expand rapidly, increasing the risk of leptospirosis (40–45). The risk of transmission can be compounded by significant precipitation and/or flooding, leading to increased contact with contaminated water and soil (37, 38, 46–55). Regions with high rates of precipitation that overlap with animal reservoir populations are at a higher risk of maintaining leptospirosis transmission to humans (15).

Countries that fall within regions of the Caribbean, Oceania, and Southeast Asia have the highest annual estimated leptospirosis morbidity rates of 50.68 (CI_95_: 14.93–87.58), 150.68 (CI_95_: 40.32–272.29), and 55.54 (CI_95_: 20.32–99.53) cases per 100,000, respectively (15). All of these regions contain tropical islands that have been shown to be vulnerable to outbreaks of leptospirosis and this may be linked to climate factors like temperature and rainfall (56). Recently, high rates of leptospirosis were correlated to annual rainfall patterns in the US state of Hawaiʻi located within the central Pacific Basin (57). In the South Pacific, annual increases in rainfall have also been shown to correlate with leptospirosis incidence in Tahiti, Fiji, and New Caledonia (56). Historical rates of leptospirosis have reported up to 975 cases per 100,000 in the rural town of Bourail on the main island of New Caledonia (58). The Seychelles in the Indian Ocean have also reported high case rates of leptospirosis up to 100.9 cases per 100,000 that may also be correlated to annual rainfall (59). While there is some information on the impact of leptospirosis across tropical island environments, there are still regions that have few reports describing the contemporary burden of this neglected tropical disease.

Sri Lanka is a tropical island nation located in the Bay of Bengal where leptospirosis is considered endemic. Leptospirosis became notifiable in the country in 1991 and modest increases in case counts were observed until 2007 (60). Between 2007 and 2008 there was a substantial increase in nationwide reported case rates from 10.99 to 37.4 cases per 100,000 suggesting a potential outbreak of leptospirosis in 2008 (60). A similar trend was observed 8 years prior in Thailand where there was a rapid increase in reporting of leptospirosis cases (61). In Thailand, there were around 200 cases of leptospirosis reported annually up until 1996, with a significant increase to 14,285 cases in 2000, followed by a decrease to 2,868 cases in 2005 (61). Flooding is associated with leptospirosis in Thailand highlighting the importance of rainfall for this disease (62). The rapid increase in leptospirosis disease in Sri Lanka over a short time period, echoes the trend observed in Thailand. However, a detailed analysis of spatiotemporal trends in Sri Lanka has not yet been undertaken. To better understand the spatiotemporal dynamics of this disease in Sri Lanka, reported cases of human leptospirosis were analyzed to identify regions of increased incidence from 2007 to 2024 in the context of precipitation.

## Materials and methods

### Reported cases of human leptospirosis in Sri Lanka, 2007–2024

Leptospirosis is a notifiable disease in Sri Lanka. To investigate the trends in leptospirosis over the last 18 years, we used publicly available weekly reported case counts published by the Epidemiology Unit of the Sri Lanka Ministry of Health (63). Weekly case counts were tabulated by administrative district for each year. Annual case counts were calculated by summation of the weekly case counts.

### Analysis and mapping of case rates

Case rates were mapped across administrative districts to better understand spatial and temporal distribution of leptospirosis in Sri Lanka. District level Geographic Information System (GIS) data was obtained from Humanitarian Data Exchange of the United Nations Office for the Coordination of Humanitarian Affairs^1^ on November 20, 2024. District level population data was extracted from the WorldPop database^2^ that was accessed on December 16, 2024. Unconstrained data at a 100-m resolution for Sri Lanka was used to determine the population of each district on an annual basis from 2007 to 2020. Due to the lack of availability of the 2021–2024 population data at the time of analysis, the 2020 population data was used for years 2021–2024. One-way ANOVA with multiple comparisons showed no significant changes in population from 2007 to 2020. Raw, empirical Bayesian smoothed (EBS), and spatially empirical Bayesian smoothed (SBS) case rates were calculated using GeoDa v. 1.22.^3^ This analysis showed smoothing had little effect on the case rates and raw rates are reported throughout the manuscript. Annual case rates were calculated by dividing the sum of all case counts for a given year by the annual population. The percent of the annual case rate was calculated by dividing the weekly case rate by the annual case rate and multiplying by 100. Cumulative rates from 2007 to 2012, 2013 to 2018, and 2019 to 2024 were calculated using the average population over the same time frame. For the time block 2019–2024, the average population of 2019 and 2020 was used to calculate rates. All choropleth maps were generated in QGIS v 3.34.3-Prizen.^4^

### Analysis of precipitation

Precipitation data was accessed from the CHIRPS: Rainfall Estimates from Rain Gauge and Satellite Observations database on March 5, 2025 (64). Daily, monthly, and annual precipitation estimates at a 0.05° resolution were used for various analysis as indicated throughout the study. Mean precipitation estimates at the national and district level spatial scales were extracted using QGIS. Annual precipitation and case rate data sets were both determined to be non-normally distributed via the Shapiro–Wilk test for normality (*p* < 0.0001) and therefore a Spearman’s Rank Correlation was used to determine if there was an association over the study period.

### Local indicators of spatial association

Local indicators of spatial association (LISA) analysis was used to identify any spatial associations within a dataset as previously described (65). This analysis calculates the local Moran’s I statistic that is designed to reject the null hypotheses of spatial randomness. This method uses computational permutations to determine a pseudo-*p*-value and determine if any spatial associations are random or significant. Significant clusters are classified as hot spots (high-high clusters), cold spots (low-low clusters), or outliers (high-low or low-high clusters). Raw rates were used to calculate the Local Moran’s I statistic in GeoDa version 1.22, as previously described (66–69). We used a first order queen contiguity and 99,999 computational permutations to determine the pseudo-*p*-value. A cut off value of *p* < 0.01 was used to determine statistical significance of any identified clusters.

### Collection of animal kidneys and genomic DNA extraction

Peri-domestic small mammals were trapped in dry and wet agricultural lands, rice storage facilities, and nearby households in the Kurunegala District, Sri Lanka, from September 2022 to March 2023, using rat and mice traps (Tomahawk, Romax Snap-R Rat Traps and Romax Snap Mouse Traps). The timing of sampling coincided with north-east monsoon season during the “Maha” rice cultivation period. Kidney tissue samples were collected from trapped *Rattus rattus* (*n* = 43), *Mus* spp. (*n* = 46), *Bandicota bengalensis* (*n* = 28), *Meriones* spp. (*n* = 3), and *Suncus murinus* (*n* = 1). Kidney samples were maintained on ice in the field and transferred to −20 °C within 6 h of collection. Genomic DNA was extracted from kidney tissues using the QIAamp DNeasy Blood and Tissue Kit (QIAGEN, Hilden, Germany).

### Screening for the *lipL32* gene from kidney samples, sequencing, and phylogenetic analysis

The extracted genomic material from potential animal reservoir kidneys was analyzed using a qPCR-based molecular method targeting the *lipL32* gene, a conserved genetic marker of pathogenic *Leptospira* (70). A TaqMan PCR amplification method, previously described was used with slight modification (71). Briefly, amplification of a 242-bp fragment of the *lipL32* gene was carried out using 2 × PrimeTime™ Gene Expression Master Mix (Integrated DNA Technology, Coralville, Iowa, United States) on a BioRad CFX Connect Real-Time System with primers LipL32-45F (5′-AAG CAT TAC CGC TTG TGG TG-3′) and LipL32-286R (5′-GAA CTC CCA TTT CAG CGA TT-3′), and the probe LipL32-189P (FAM-5′-AA AGC CAG GAC AAG CGC CG-3′-BHQ1). Amplification conditions were 50 °C for 2 min, 95 °C for 3 min, and 45 cycles of 95 °C for 15 s and 58 °C for 60 s.

Phylogenetic analysis of *Leptospira* species has been acomplished using the *lipL32* gene (72, 73). In the present study, Sanger sequencing of all *lipL32* positive samples was carried out via a PCR reaction for a 450-bp region of the *lipL32* gene with primers LipL32_F (5′- ATC TCC GTT GCA CTC TTT GC-3′) and LipL32_R (5′- ACC ATC ATC ATC ATC GTC CA-3′) (74, 75). The purified 450-bp amplicons were sequenced at the Advanced Studies in Genomics, Proteomics and Bioinformatics (ASGPB) core at the University of Hawaiʻi at Mānoa and visualized using SnapGene software. The phylogenetic relationships with other *Leptospira* species were examined using the Molecular Evolutionary Genetics Analysis (MEGA) software (v12.0.8) by aligning with the *lipL32* sequences retrieved from the NCBI database (Supplementary Table S1).

#### Ethics statement

All de-identified human data used in this study were publicly available from the Epidemiology Unit of the Sri Lanka Ministry of Health and did not require Institutional Review Board approval (63). The animal work described in this manuscript was approved by the Committee for Ethical Clearance on Animal Research at the University of Peradeniya (proposal ID VERC-22-03).

## Results

### Incidence of leptospirosis in Sri Lanka, 2007–2024

**Figure 1:**
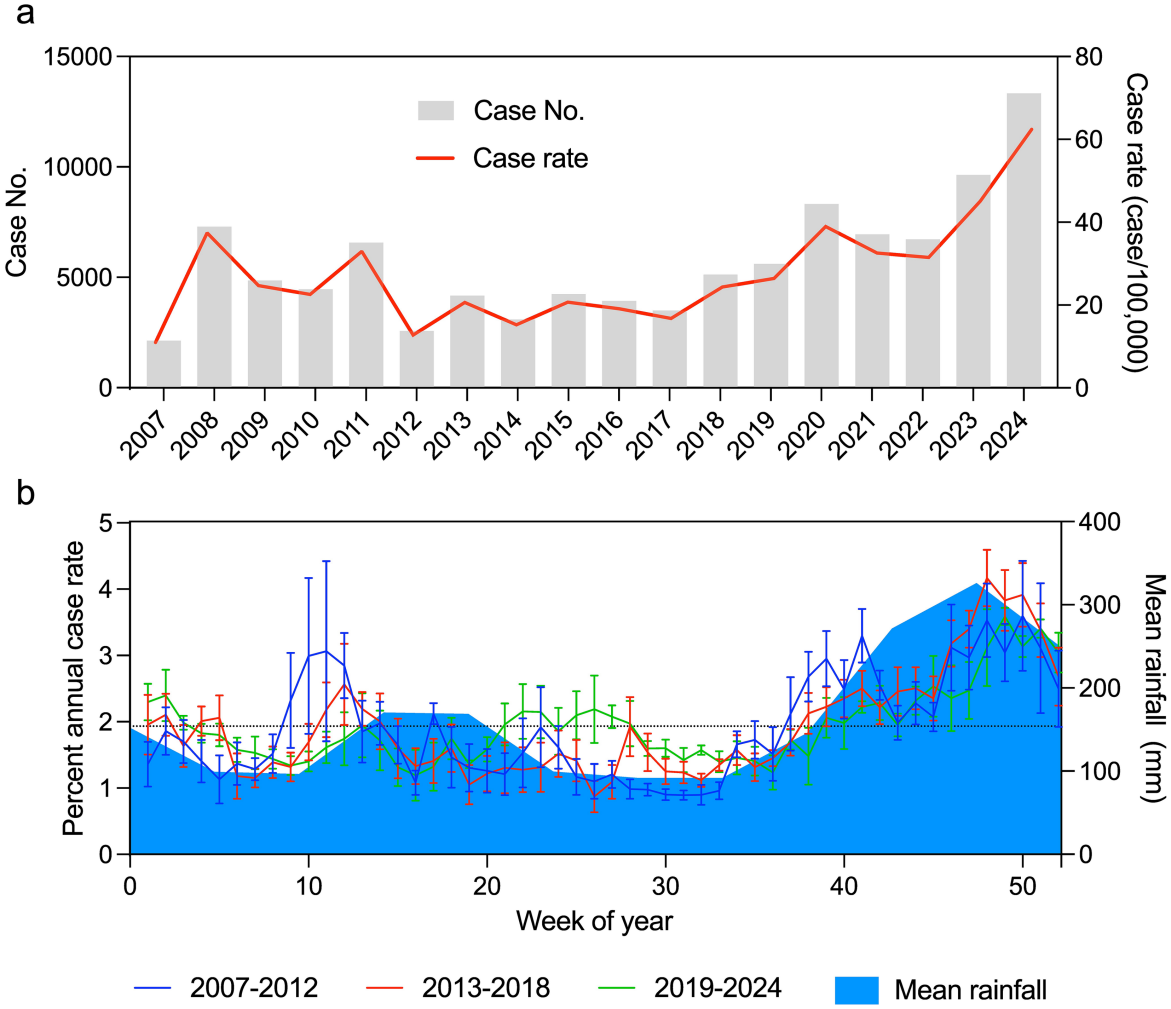
Leptospirosis in Sri Lanka from 2007 to 2024. **(a)** Case counts and rates for Sri Lanka per annum. Grey bars represent case counts and the red line represents the annual case rate. **(b)** Weekly contributions to annual case rate and mean monthly precipitation. The weekly contribution to the annual case rate is expressed as a percent of the entire year. Each line represents the mean case rate for 6 year time blocks (2007–2012, 2013–2018, 2019–2024) and the error bars represent the standard deviation. The blue solid underlay represents the mean monthly precipitation from 1981 to 2024.

In 2007 there were 2,129 reported cases of leptospirosis with a case rate of 10.99 cases per 100,000 (Figure 1a). The following year there was a significant increase with a total of 7,295 cases and a case rate of 37.40 cases per 100,000 (Figure 1a). Leptospirosis cases stabilized to an average annual case count of 4,372 (SD = 1,142) and a case rate of 21.48 (SD = 5.67) cases per 100,000 from 2010 to 2019 (Figure 1a). During this time frame there was a spike in leptospirosis disease in 2011 with a total of 6,564 cases and a case rate of 32.98 cases per 100,000 (Figure 1a). In 2020, there were 8,323 cases with a case rate of 38.99 cases per 100,000, marking the beginning of a major increase in the incidence of this neglected tropical disease (Figure 1a). There were sustained levels of leptospirosis cases in 2021 and 2022, with 6,946 and 6,719 cases with corresponding cases rates of 32.54 and 31.48 cases per 100,000, respectively (Figure 1a). In 2023, there were 9,630 cases of leptospirosis with a case rate of 45.12 cases per 100,000 (Figure 1a). The highest annual number of leptospirosis cases was recently reported at 13,328 in 2024, with a case rate of 62.44 cases per 100,000 (Figure 1a). From 2007 there was an average 2.77 (range 1.20 to 6.26, SD = 1.27) fold-increase in leptospirosis cases per year, highlighting this disease as an increasing cause of morbidity in Sri Lanka.

### Annual trends of leptospirosis and precipitation

To better understand the trends of leptospirosis disease in Sri Lanka, we tabulated weekly case rates in 6 year blocks, from 2007 to 2012, 2013 to 2018, and 2019 to 2024. We calculated the percentage of the annual case rate for each week of each year to identify increases or decreases from the baseline. An even distribution of the case rate on an annual basis would result in each week accounting for ~1.9% of the annual case rate. Any week that has a percent annual case rate below 1.9% indicates a reduced leptospirosis burden for that week in the context of the annual trend. Any week with a percent annual case rate above 1.9% indicates an increased leptospirosis burden for that particular week within that year. In general, case rates of leptospirosis were consistent across the time blocks analyzed. However, from 2007 to 2012 there was an additional increase in annual cases rates around week 10 (Figure 1b). This departure from the consistent annual trends in leptospirosis is consistent with an increased case burden in 2011 that is further discussed below. During the first 37 weeks of each year there are fluctuations between one and 3 % of the annual case rate, with the majority of weeks below the 1.9% marker (Figure 1b). At week 38, there is a steady increase in leptospirosis case rates (Figure 1b). The increase observed in the last quarter of the year is consistent across all time blocks that were analyzed indicating annual seasonality of leptospirosis in Sri Lanka during the 18-year study period (Figure 1b).

Monthly precipitation rates for Sri Lanka from 1981 to 2024 were compiled to determine if any correlation or overlap could be observed with annual weekly trends in human leptospirosis. Average monthly precipitation rates in Sri Lanka ranged from 91.86 to 326.23 mm with a mean monthly rate of 163.55 mm (SD = 79.16). From 1981 to 2024, there were two seasonal increases in monthly average precipitation. The first increase in precipitation occurs from April to May with average rates of 170.45 mm (SD = 63.15) and 168.77 mm (SD = 79.57) (Figure 1b). The second increase in monthly precipitation rates begins in September and extends to January of the subsequent year (Figure 1b). The average precipitation rates during this second seasonal increase range from 145.98 mm (SD = 58.39) in the month of September to 326.23 mm (SD = 81.94) in the month of November (Figure 1b). The increase in precipitation rates during the last months of the year correlate with the increase in human leptospirosis case rates (Figure 1b).

### Geographic distribution of leptospirosis incidence, 2007–2024

**Figure 2:**
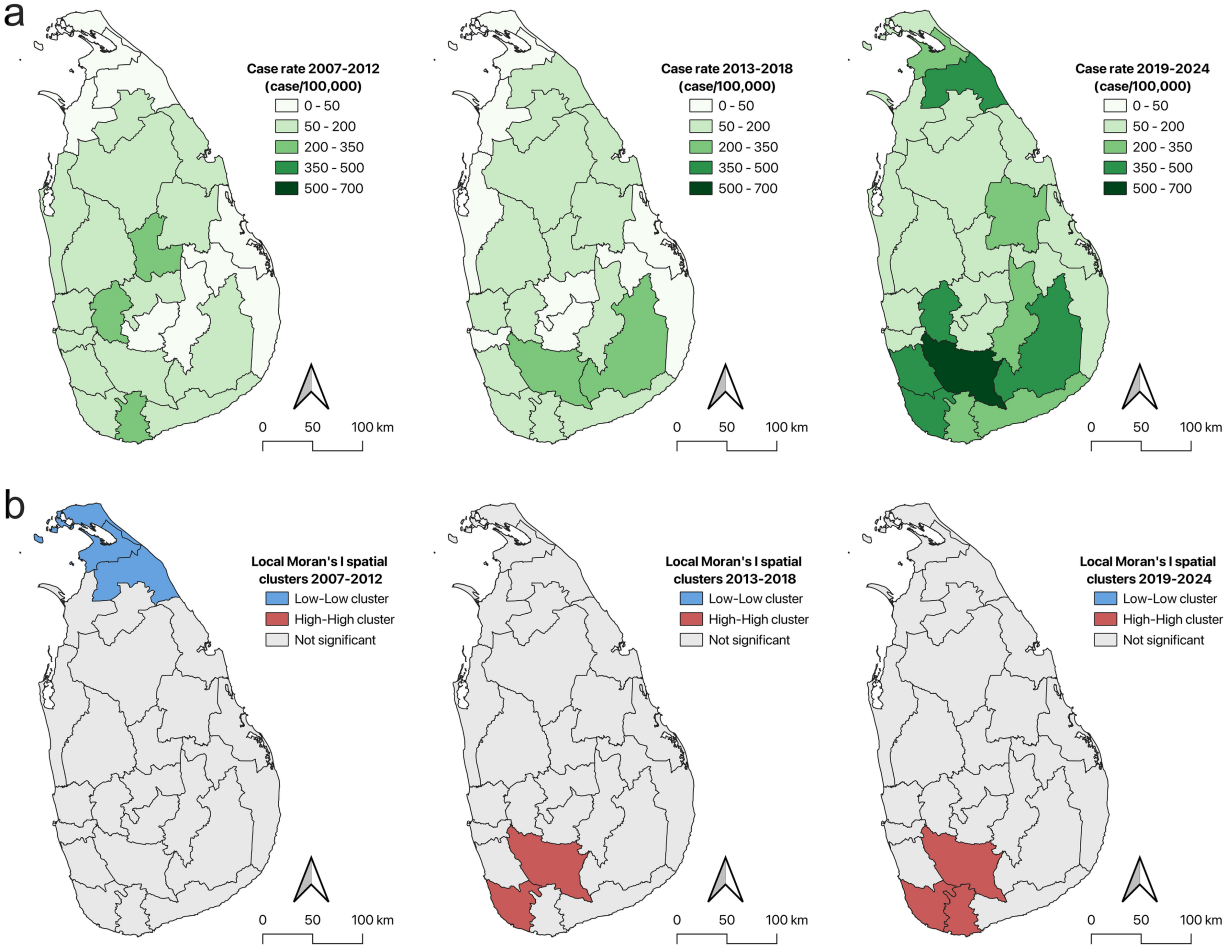
Spatial trends of leptospirosis from 2007 to 2024. **(a)** Cumulative case rates of leptospirosis in Sri Lanka from 2007 to 2012, 2013 to 2018, and 2019 to 2024 show amplification of disease and geographic spread. Case rates are expressed as cases per 100,000 population. **(b)** Spatial clusters characterized by LISA analysis identified a low-low cluster from 2007 to 2012 and a high-high cluster from 2013 to 2024.

Analysis of leptospirosis across the country requires the use of a finer resolution spatial scale. Sri Lanka is broken down into 25 administrative districts with populations ranging from ~54,000 to ~2,460,000. Due to the large variation in population between districts we analyzed the cases rates using various methods of smoothing. We identified minimal influence on case rates from EBS or SBS smoothing at the district level, and therefore are reporting the raw case rates throughout the manuscript (Supplementary Figure S1). There was a steady increase in case rates and distribution of leptospirosis cases from 2007 to 2024 (Supplementary Figures S2, S3). The highest case rate observed in any district in 2007 was 35.83 cases per 100,000 while a district in 2024 peaked at 188.2 cases per 100,000 (Supplementary Figure S3). In 2007 the burden of leptospirosis was consolidated in the southern regions, but by 2024, there was an increase in case rates throughout the entire country, with several northern and southern districts having case rates in excess of 140 cases per 100,000 (Supplementary Figure S2). When analyzed in 6 year time blocks, increases are also observed in case rates and distribution of leptospirosis (Figure 2a). Cumulative case rates ranged from 0.9 to 282.55 cases per 100,000 between 2007 and 2012 with lower case rates observed in the northern and eastern districts (Figure 2a). Between 2013 and 2018, case rates ranged from 20.16 to 245.32 cases per 100,000, with focal regions of disease consolidated in southern districts (Figure 2a). In the final time block between 2019 and 2024, cumulative case rates ranged from 60.09 to 668.03 cases per 100,000 with clear geographic expansion to northern districts in the country (Figure 2a). Geographic spread of leptospirosis was observed over the study period showing expansion to northern districts and increases in case rates throughout the country.

LISA analysis through the local Moran’s I statistic was used to identify any significant spatial clusters using the cumulative raw case rates for each district. Between 2007 and 2012 there was a low-low cluster identified in the northern districts of the country indicating a region of lower leptospirosis incidence (Figure 2b). This low-low cluster included districts Kilinochchi, Mullaitivu, and Jaffna with pseudo *p* values of 0.00529, 0.00683, and 0.00728, respectively. This low-low cluster was not identified between 2013 and 2018, while a high-high spatial cluster can be observed centralized around two districts in the south during this period (Figure 2b). The center of the high-high cluster included districts Ratnapura and Galle with pseudo *p* values of 0.00271 and 0.00642, respectively. The center of the high-high cluster expanded to include three southern districts between 2019 and 2024 (Figure 2b). However, the expansion of the high-high cluster observed between 2019 and 2024, did not expand the total extent of this hot spot because the total number of first order neighboring districts remains at ten. The high-high cluster during this time frame centered around districts Ratnapura, Galle, and Matara with pseudo *p* values of 0.0012, 0.00673, and 0.00684, respectively. This analysis identified spatial and temporal shifts in leptospirosis disease cluster patterns in Sri Lanka when analyzed in 6 year time blocks.

### District level correlation identified between leptospirosis incidence and annual rainfall

**Figure 3:**
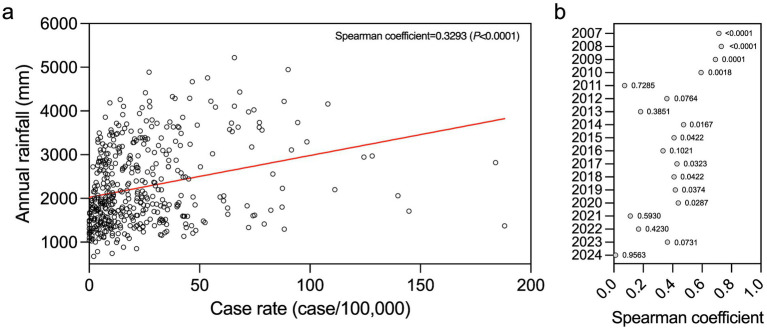
Correlation of annual rainfall and case rate at the district level. **(a)** The annual rainfall and the annual case rate for each district is plotted showing a correlation between the two parameters. Each point represents rainfall and case rate parameters for one district for 1 year. The Spearman coefficient for all points is 0.3293, *p* < 0.0001. **(b)** Correlation of rainfall and case rate broken down by year. The point indicates the Spearman coefficient of correlation for each year and the *p* value is presented immediately to the right side of the point.

Annual precipitation appears to drive seasonal increases in leptospirosis in Sri Lanka (Figure 1b). We analyzed the mean annual rainfall at the district level to characterize any correlation to leptospirosis disease (Supplementary Figures S4, S5). Increased annual precipitation levels can be observed consistently in the southern districts (Supplementary Figure S4). Analysis of rainfall from year to year across all districts showed relatively consistent trends of precipitation (Supplementary Figure S5). To determine if there was a correlation at the district level, we analyzed the mean annual rainfall and compared to the annual case rate of each district (Figure 3a). A weak yet statistically significant positive correlation (Spearman coefficient = 0.3293, *p* < 0.0001) was identified between rainfall and case rate from 2007 to 2024 (Figure 3a). When this analysis is broken down per annum, positive correlations were observed in 2007, 2008, 2009, and 2010, with corresponding Spearman coefficients of 0.7145, 0.7306, 0.6909, and 0.5931 (Figure 3b). In the following years, a decreasing trend was observed in the correlation of annual rainfall and case rates by district (Figure 3b). In 2011 there was no correlation of annual rainfall and case rates at the district level (Spearman coefficient = 0.0731, *p* = 0.7285, Figure 3b). No correlation was observed in 2024 as well with a Spearman coefficient of 0.0115 (*p* = 0.9563, Figure 3b). These deviations may indicate that finer resolution temporal dynamics and environmental trends need to be investigated to understand the role of rainfall on leptospirosis incidence in these time frames. Other possible reasons that may drive these deviations are discussed in the following sections. In general, over the 18-year study period, a positive correlation between annual rainfall and leptospirosis case rate was identified at the district level in Sri Lanka.

### Outbreak of leptospirosis in February and March, 2011

**Figure 4:**
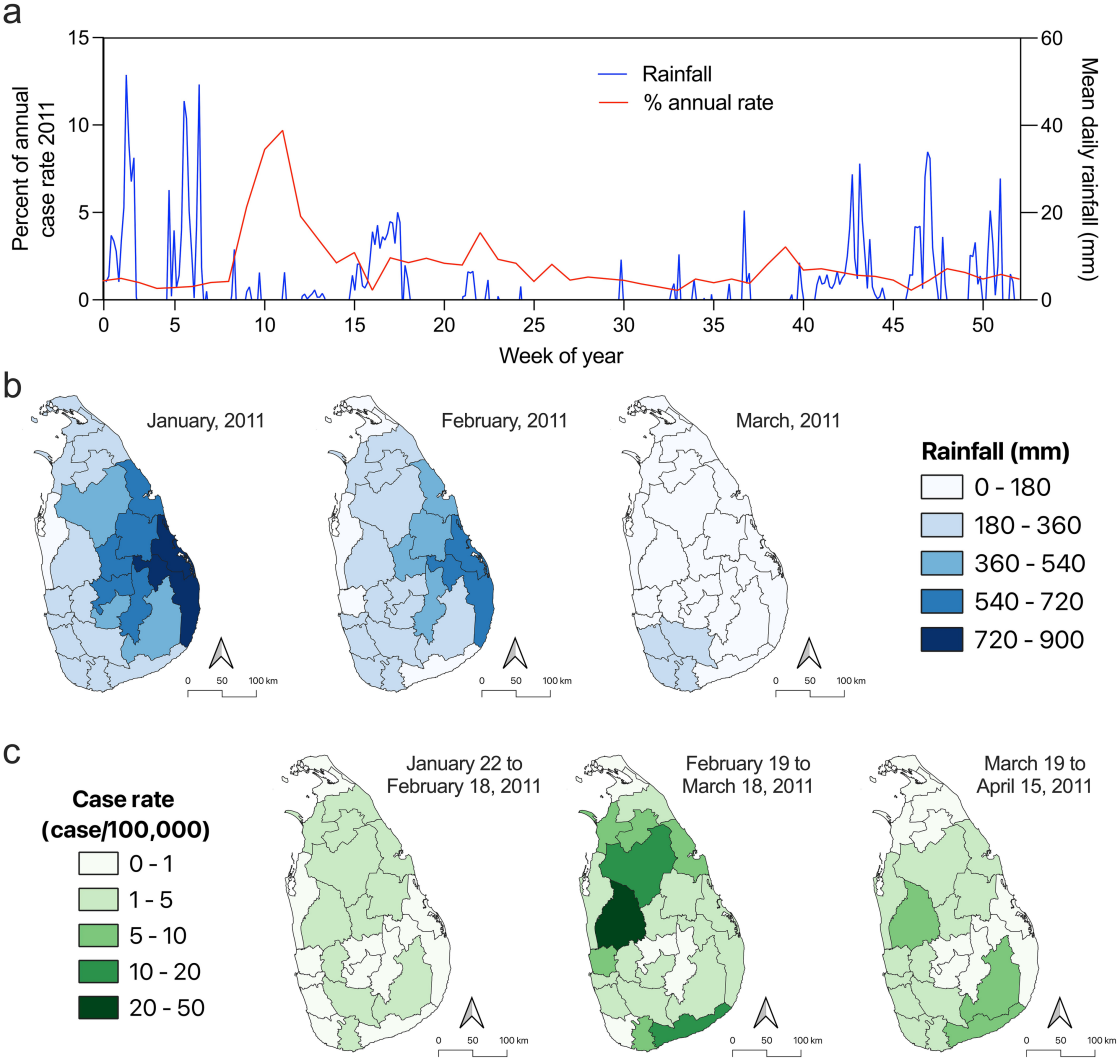
Analysis of leptospirosis and rainfall in 2011. **(a)** Plot of mean daily rainfall at the national level (blue line) compared to percent annual case rate of leptospirosis for 2011 (red line). Major rainfall events occurred in the middle of January and early in February of 2011 leading to major flooding (79, 80). **(b)** Mean rainfall in each district for the months of January, February, and March in 2011 showing increased precipitation in the early months of the year. **(c)** Cumulative rates of leptospirosis over the indicated time periods showing a spike in cases between February 19 to March 18, 2011. Rate maps in **(c)** are staggered based on the national trend of leptospirosis rates in **(a)** to highlight the lag of disease reporting until the middle of February.

The year 2011 showed the second lowest Spearman correlation coefficient between rainfall and case rate, prompting a more detailed examination of this period. In the 2007–2012 period, the mean case rate spike during the first 15 weeks of the year had large standard deviations (Figure 1b). Initially, no correlation was identified between precipitation and reported leptospirosis over the entirety of 2011. However, isolation of the percent annual case rates of 2011 show a significant spike in leptospirosis disease around week ten that accounted for the anomaly identified in Figure 1b. The spike in leptospirosis that occurred between week eight and week fourteen accounted for 35% of the 2011 annual burden of disease (Figure 4a). Several days exceeded 40 mm in mean daily rainfall over the first 6 weeks of the year prior to the spike in leptospirosis cases (Figure 4a). Dissection of monthly precipitation at the district level show an increase in rainfall along the east coast and central region of Sri Lanka in January and February, 2011 (Figure 4b). Weekly case rates of leptospirosis at the district level ranged from 0 to 4.61 cases per 100,000 between January 22 and February 18, 2011 (Figure 4c). Between February 19 and March 18, 2011 there was an increase in case rates up to 49.08 cases per 100,000 (Figure 4c). Following this, mean daily rainfall was reduced in the month of March and did not exceed 40 mm at any time in the remainder of the year when case rates reduced to a range of 0 to 8.66 cases per 100,000 from March 19 to April 15, 2011 (Figures 4b,c). While we saw no evidence of correlation between annual rainfall and district level case rates in 2011 (Figure 3b), a finer resolution analysis of temporal precipitation trends and case rates suggest that there was a temporal lag in case rates after a period of heavy rainfall in the early months of 2011.

### Trends of leptospirosis and precipitation in 2024

**Figure 5:**
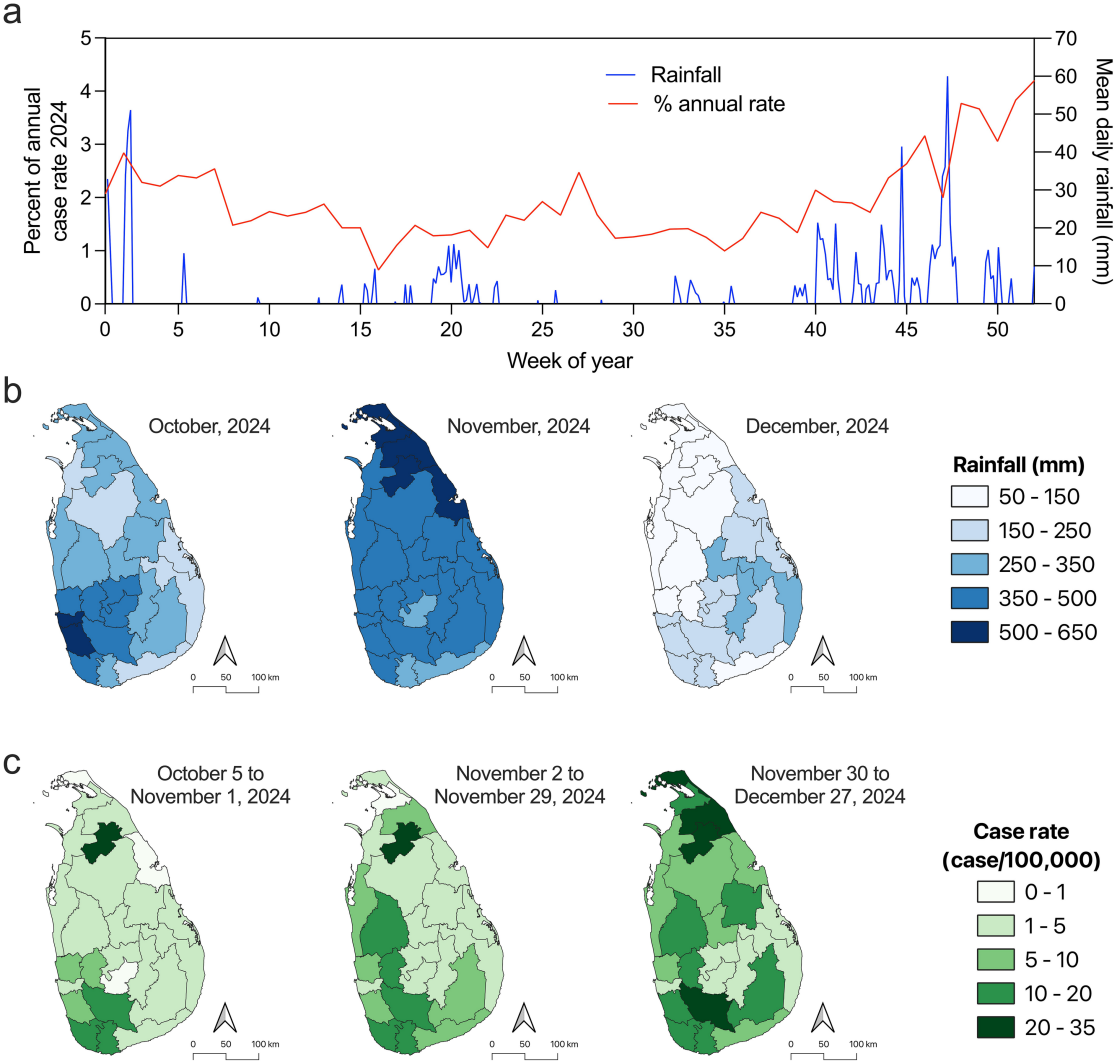
Analysis of leptospirosis and rainfall in 2024. **(a)** Plot of mean daily rainfall at the national level (blue line) compared to percent annual case rate of leptospirosis for 2024 (red line). **(b)** Mean rainfall in each district for the months of October, November, and December in 2024 showing increased precipitation in November. **(c)** Cumulative rates of leptospirosis over the indicated time periods showing an increase in cases in northern districts that may be associated with increased rainfall in November, highlighting a temporal lag of disease reporting from prior increased precipitation.

The highest number of annual recorded cases of leptospirosis occurred recently in 2024 with 13,328 cases and a case rate of 62.44 cases per 100,000 (Figure 1a). Similar to 2011, no obvious correlation was identified between annual rainfall and district level case rates at the national level, encouraging further analysis. In the first 35 weeks of 2024, the average weekly contribution to the leptospirosis burden over the year was 1.65% (range 0.64 to 2.84%) (Figure 5a). From week 36 to the end of the year the average contribution to disease increased to 2.49% (range 1.23 to 4.19%) (Figure 5a). An increase was also observed in the mean daily rainfall across the country that coincided with the increased burden of case rates throughout the year (Figure 5a). This trend is similar to the trend observed over the 18 years study period identifying coincidence of increased rainfall and leptospirosis disease (Figure 1b). In October 2024 there was higher precipitation identified in the southern districts, while there was a nationwide increase in November 2024 (Figure 5b). In December 2024, there was a major drop off in the amount of rain throughout the country (Figure 5b). Leptospirosis case rates ranged from 0 to 20.29 per 100,000 from October 5 to November 29, 2024 (Figure 5c). The mean district level case rate was 4.83 from October 5 to November 1, and increased to 6.83 between November 2 and 29 (Figure 5c). From November 30 to December 27, 2024 there was a marked increase with rates ranging from 1.53 to 33.2 cases per 100,000 with a district level average rate of 11.86 (Figure 5c). Similar to 2011, there was no evidence supporting a correlation between annual precipitation and district level case rates in 2024 (Figure 3b). However, analysis of finer resolution temporal dynamics of rainfall and case rates show a potential relationship in 2024.

### Detection of pathogenic *Leptospira* species in animal reservoirs during the 2022–2023 north-eastern monsoon season

**Figure 6:**
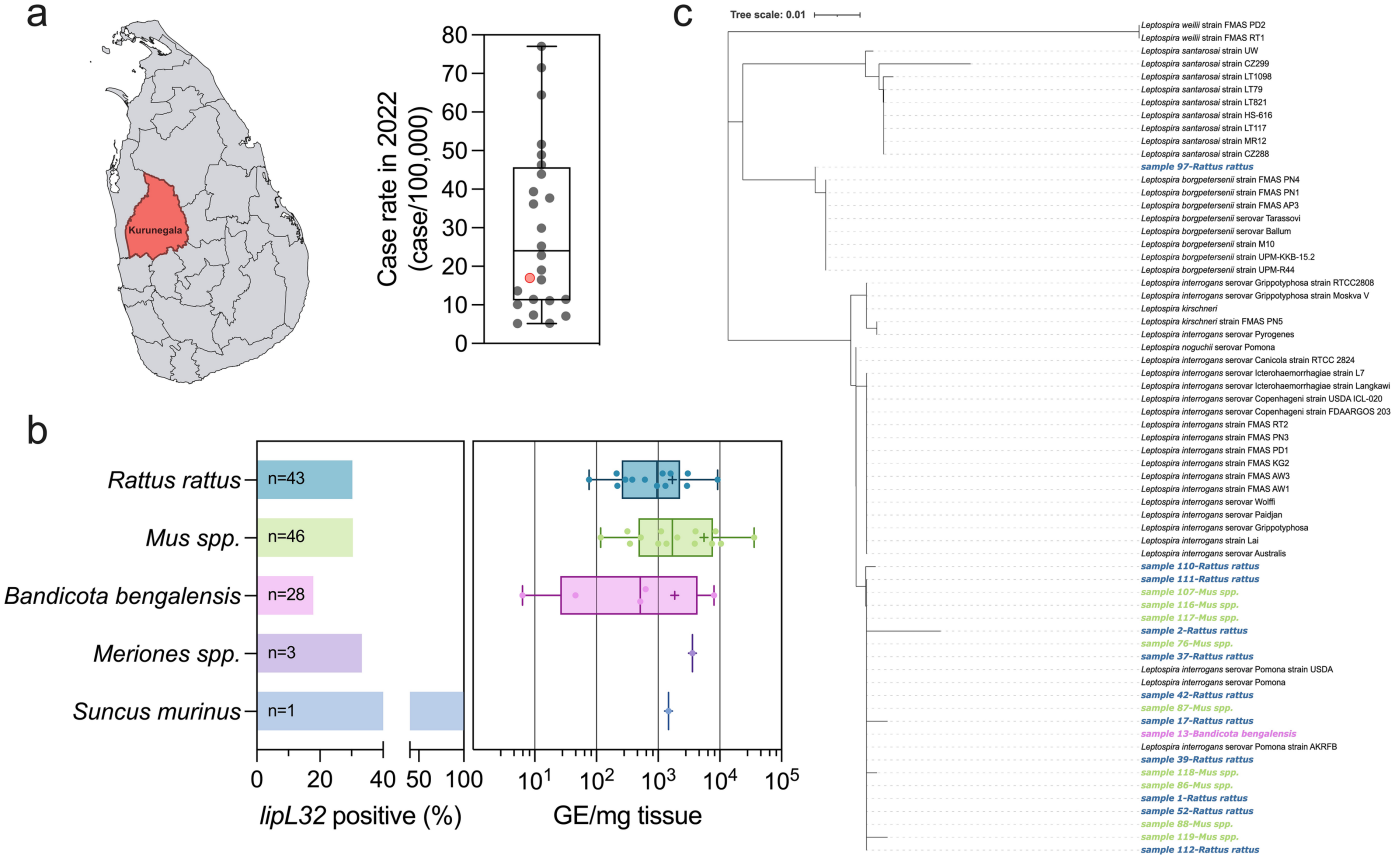
Identification of *lipL32* positive animals in Kurunegala district. **(a)** Animal kidneys were isolated from Kurunegala district (red) in 2022. Annual case rates for reported human leptospirosis for 2022 is shown in the box plot. The red dot represents the case rate of Kurunegala district in 2022. Whiskers on box plots represent minimum value to maximum value and dots represent all values in data set (*n* = 25). **(b)** A total of 121 kidney samples were tested from potential animal reservoirs and 34 were *lipL32* positive. Distribution of positive percentages of individual animal reservoir species is shown in the left panel. The right panel represents genomic equivalents (GE) per mg of kidney tissue. Positive *lipL32* kidney samples in box plots and bar graph: *R. rattus* samples (blue, *n* = 13), *Mus* species samples (green, *n* = 14), *B. bengalensis* (magenta, *n* = 5), *Meriones* species samples (purple, *n* = 1), and *S. murinis* samples (light, *n* = 1). Colors correlate to panel **(c)**. Whiskers on box plots represent minimum value to maximum value and dots represent all positive *lipL32* samples for each data set. The plus sign represents the mean of the corresponding data. (c) Twenty-one *lipL32* genes were sequenced from *R. rattus* (blue, *n* = 11), *Mus* spp. (green, *n* = 9), and *B. bengalensis* (magenta, *n* = 1) and compared to other sequenced pathogenic *Leptospira* strains (Supplementary Table S1) showing similarity to *L. borgpetersenii* (*n* = 1) and *L. interrogans* (*n* = 20).

To investigate the presence of pathogenic *Leptospira* in animals, kidney samples from potential reservoirs were screened for the presence of the *lipL32* gene unique to pathogenic *Leptospira* species (Figure 6a). Over 2022, Kurunegala district reported 17.01 cases of leptospirosis per 100,000 (Figure 6a). A total of 121 kidneys were collected from various mammals including *Rattus rattus* (*n* = 43), *Bandicota bengalensis* (*n* = 28), *Mus* spp. (*n* = 46), *Meriones* species (*n* = 3), and *Suncus murinus* (*n* = 1) from the Kurunegala district of Sri Lanka (Figure 6b). Overall, thirty-four kidneys (28.1%) were positive for *lipL32* across all samples tested (Figure 6b). *Mus* species and *R. rattus* showed high percent positivity for *lipL32* overall with (13/43) 30.4% and (14/46) 30.2%, respectively (Figure 6b). Five out of 28 (17.8%) *B. bengalensis* kidney samples were positive for *lipL32* gene. Among *Meriones* spp., one out of three samples (33.3%) tested positive. The single *S. murinus* kidney sample collected in this study also tested positive. Positive kidney samples from *R. rattus* had a range of 75.77 to 9,224 genomic equivalents (GE) per mg of tissue with a mean of 1,695 GE/mg (Figure 6b). The mean GE/mg of tissue from *B. bengalensis* kidney samples was slightly higher at 1,857 GE/mg, ranging from 6.35 to 8,089 (Figure 6b). The single *S. murinus* sample tested was positive and had 1,482 GE/mg of kidney (Figure 6b). The single *lipL32* positive *Meriones* sample was ~2x the *B. bengalensis* samples with 3,616 GE/mg of kidney (Figure 6b). *Mus* species had the highest mean kidney carriage with a mean GE/mg of tissue at 5,527, ranging from 116.7 to 36,090 (Figure 6b). The *lipL32* gene from 21 of 34 positive samples was sequenced and compared with other pathogenic *Leptospira* strains (Supplementary Tables S1, S2) by phylogenetic analysis. A single sequence from a *R. rattus* kidney was closely related to *L. borgpetersenii* (Figure 6c). The remaining 20 *lipL32* sequences from *R. rattus* (*n* = 10), *Mus* species (n = 9), and *B. bengalensis* (n = 1) clustered with *L. interrogans* (Figure 6c).

## Discussion

Leptospirosis is an important zoonotic infection that causes significant morbidity globally, disproportionately affecting tropical climates, including the country of Sri Lanka (15). The nature of leptospirosis disease is complex and transmission is influenced by many factors including temperature, rainfall, animal reservoirs, and human behavior making it a major global concern. In the present study, we sought to better understand the temporal and spatial dynamics of leptospirosis disease and its relationship with precipitation in Sri Lanka. After the 2007–2008 spike in leptospirosis in Sri Lanka, there was an increase in reported disease that peaked recently in 2024 with 13,328 cases. These increases may be due to geographic spread from many factors including animal reservoirs, improved clinical awareness, and increased surveillance. Previous studies have highlighted the hyperendemicity of leptospirosis in Sri Lanka. Our study confirms this while providing additional context to the temporal dynamics of incidence and geographic spread (60, 76, 77).

The largest year after year increase in case counts was identified between 2007 and 2008. This increase was also previously reported, and the main finding of this study suggested that a potential emergence event occurred in 2008 (60). Between 2009 and 2019, case counts and rates were relatively stable at an elevated rate compared to 2007, further supporting the 2008 emergence hypothesis. In 2020, there was another significant increase in case counts and the highest reported case rate, exceeding the rate reported in 2008 (76). Authors of this study highlight that this was one of the largest outbreaks of leptospirosis in Sri Lanka, but was overshadowed by the COVID-19 pandemic (76). Case counts and case rates climbed even higher in 2023 and 2024 suggesting that there was significant spread of leptospirosis throughout the country possibly due to nationwide increases observed several years prior. Analysis of weekly case rates showed consistent patterns of disease over the 18 years analyzed in the current study. In general, the highest rates of leptospirosis were associated with the final quarter of the year correlating with an increase in monthly precipitation. For simplicity, we analyzed these trends in 6 year blocks. The 2007–2012 block showed a significant departure from the rate due to increased leptospirosis burden in 2011. Exclusion of the 2011 rates from this analysis reduces the mean of the percent annual case rate for the 2007–2012 time frame to comparative levels with the other time blocks. At the national level, this data shows that there is a consistent trend between leptospirosis disease and increased precipitation, suggesting that seasonal climatic conditions may drive leptospirosis disease in Sri Lanka.

In the 2007–2012 time block, leptospirosis was dispersed throughout the country with some southern and central districts having higher case rates. No significant high-high LISA spatial clusters were identified during this time frame but a low-low spatial cluster was identified centered around the three northern districts of Jaffna, Kilinochchi, and Mullaitivu. This suggest that these northern districts and their spatial neighbors had reduced leptospirosis disease compared to the districts in the south during this early time frame. Between 2013 and 2018, cumulative rates were highest in several southern districts and a high-high spatial cluster was identified around the southern districts of Galle and Ratnapura. The low-low cluster in the northern districts disappeared during this time frame, suggesting that leptospirosis disease in this region was no longer significantly lower than other regions throughout the country, supporting the notion that geographic spread was underway. The district of Ratnapura had the highest cumulative rate between 2019 and 2024 with 668 cases per 100,000 population. Five districts including Kalutara, Mullaitivu, Galle, Kagalle, Monaragala had very high case rates ranging from 361 to 459 cases per 100,000. The district of Mullaitivu was within the low-low spatial cluster from 2007 to 2012, but from 2019 to 2024 showed extremely high case rates further supporting the geographic spread of leptospirosis to northern districts over the 18-year period of the study. LISA analysis during the final time frame showed no low-low spatial clusters but did show an expanded high-high cluster in the southern districts. The analysis of leptospirosis at the district level identified geographic spread and amplification of case rates from 2007 to 2024.

National level coincidence of leptospirosis disease and precipitation rates prompted us to analyze seasonality at a finer resolution spatial scale. We compared annual case rates to annual precipitation at the district level. This analysis identified that over the 18-year period there was a weakly positive correlation between case rate and precipitation further suggesting that annual rainfall contributes to leptospirosis in Sri Lanka. This result agrees with other work recently published showing a positive correlation with rainfall from 2011 to 2022 (78). While this correlation is statistically significant when comparing all data points over the 18-year period, when the analysis is broken down by year it is only significant in the years 2007, 2008, 2009, 2010, 2014, 2015, 2016, 2017, 2018, 2019, and 2020. There is a reduction in the annual correlation between precipitation and case rate that can be observed from a Spearman coefficient of 0.7145 in 2007 to a Spearman coefficient of 0.01154 in 2024, signifying possible changes that occurred over the course of the 18-year study period. The decreasing trend in correlation can be explained by the occurrence of outbreaks, misdiagnosis, and/or saturation of leptospirosis across the country.

Outbreaks could obscure the observed correlation due to a rapid increase in cases over a short time period, as was observed during a flood-associated outbreak in 2011 (77). In 2011, there was no statistically significant correlation between case rate and rainfall in districts when looking at the annual scale justifying a more in-depth analysis. However, when case rates are broken down over the course of 2011, there was significant outbreak starting in February accounting for 35% of the reported leptospirosis cases for the year. Prior to this outbreak there were two significant increases in precipitation in January and February associated with major floods that affected over 1 million people, leaving at least 37 people dead (79, 80). The rainfall event that occurred in the middle of January 2011 was not associated with an immediate temporal lag in reported leptospirosis. However, the lack of reported cases may be attributed to the second major rainfall event in early February disrupting disease reporting. After the rainfall event in early February there was an associated increase in reported leptospirosis cases with a 2 week temporal lag that likely represents cases associated with both flooding events. These precipitation events caused major damage and likely contributed to the significant increase in leptospirosis cases seen immediately after the flood events and further supports the correlation between rainfall and disease contrary to the annual analysis. In 2024 there was also no correlation between annual rainfall and disease incidence at the annual scale. The overall trend of leptospirosis disease for the entire country in 2024 followed the same trend identified over the 18-year study period. Increases in leptospirosis at the end of the year in the southern and norther districts was preceded by increased rainfall, further indicating the correlation between precipitation and disease. During 2024 there were elevated leptospirosis cases that do not appear to be directly linked to major nationwide rainfall events.

Another explanation for the temporal decrease in the correlation between rainfall and case rates could be overreporting of leptospirosis cases. As was observed in this study, there was a major increase in cases reported from 2007 to 2024 and this trend is inverse to the correlation between rainfall and case rate. While we believe that this is not likely the case, it is possible due to the consistency observed in weekly case rate contributions over the 18-year period. The final possibility is that after the potential emergence of leptospirosis in Sri Lanka that was previously documented (60), leptospirosis has spread geographically and established across the country in animal reservoirs that maintain the disease. This would help explain the increase in cases overtime, the geographic spread, and, in part, the reduction of the correlation between rainfall and case rate. In this scenario, the presence of leptospirosis in animal and environmental reservoirs at high concentrations would increase the likelihood of transmission independent of environmental stresses like the major flood events in 2011. For this reason it is critical to understand how animal reservoirs contribute to maintenance of leptospirosis in the environment leading to indirect transmission to humans in Sri Lanka.

We finally looked at contemporary presence of pathogenic *Leptospira* in animal reservoirs within the district of Kurunegala in 2022. Kurunegala district had 17.01 cases/100,000 of reported human leptospirosis and was in the interquartile range of all districts for 2022. Kidney samples that are positive for *lipL32* represent the potential for an animal to be excreting pathogenic *Leptospira* into the environment. All potential animal reservoir species tested had at least one *lipL32* positive sample indicating that they likely contribute to environmental contamination with pathogenic *Leptospira*. This also suggests that numerous maintenance reservoirs, beyond what was tested presently, may contribute to environmental spread of leptospirosis. Sequence analysis of the *lipL*32 gene identified that *L. borgpetersenii* and *L. interrogans* were identified, consistent with other reports from animal reservoirs tested in Sri Lanka (81). Overall, there was a 28.1% positivity rate across all animals tested with the majority of positives from *Mus* species and *R. rattus*, both known reservoirs of leptospirosis. *Mus* species had the highest level of renal carriage, with a mean renal carriage rate 3x what was observed in *R. rattus*. Other work surveying animals in tropical islands has identified similar trends. In Hawaiʻi, a survey of small mammals from 1990 to 2003 identified that 18.2% of animals tested (*n* = 15,171) were culture positive for *Leptospira*, and the serogroup Icterohaemorrhagiae was the most prevalent (26). A survey of 12 potential animal reservoirs on Reunion Island in the Indian Ocean identified high seroprevalence (13.2–79.5%) in all species tested and high *lipL32* positivity (15.7–84.6%) in all but one species (82). Similar to our observations in Sri Lanka, *Mus* species had the highest *lipL32* positivity followed by *R. rattus* further indicating that these species are critical maintenance reservoirs in tropical island settings (82). A survey of *Mus musculus*, *R. rattus*, *R. norvegicus*, and *Herpestes auropunctatus* from cattle farms in Puerto Rico also identified that 38% of the surveyed animals were positive for *lipL32* with species specific rates ranging from 13 to 59% (83). The rates of renal carriage observed in animal reservoirs in Sri Lanka are comparable to other areas with increased rates of leptospirosis disease.

The work presented here identifies national level spatiotemporal trends in human leptospirosis in conjunction with environmental patterns and a snapshot of renal carriage of pathogenic *Leptospira* in animal reservoirs in the central region of Sri Lanka. This work is valuable, but not without limitations. The case data is based on reports to the Epidemiology Unit of the Sri Lanka Ministry of Health and could over or under represent actual leptospirosis in the country. There is significant symptomatic overlap with other febrile illnesses, including dengue fever and malaria, that could lead to misdiagnosis, thereby obscuring the real number of cases that are reported (10–14). The animal reservoirs tested only covered a small spatial and temporal range limiting the probative potential of this work. From 2007 to 2024, there was an increase of human leptospirosis in the northern districts but we do not know how animal reservoirs contributed to this spread. Environmental information (i.e., precipitation) was able to help explain seasonal changes in case rates but does not help us understand the entire extent of geographic spread across the nation over 18 years, an aspect that could be explained by temporal examination of animal reservoirs if data were available. The correlation identified between precipitation and case rates in Sri Lanka does stand in agreement with other pivotal work done on climate driven models of leptospirosis in tropical oceanic basins and other regions with high rates of disease (56, 84). Another limitation of our work is the spatial resolution of the precipitation data used for analysis in conjunction with the use of administrative boundaries may not be ideal. A more finetuned analysis of cases at a higher resolution spatial scale would likely tease apart the role of precipitation and may help explain the decreasing positive correlation we observed. Some factors that may also contribute to the decrease in correlation between case rates and precipitation over time may include changes to reporting procedures, healthcare access, agricultural practices, or public health outreach. Other location specific factors outside of climate are likely contributing to the seasonality of leptospirosis globally including population density, behavior, and presence of maintenance reservoirs. Our results appear to agree with that hypothesis, as observed with the decreasing correlation of precipitation and case rate over 18 years. Taken together, this highlights the need for increased animal surveillance in parallel with environmental and human disease surveillance to truly understand how leptospirosis travels across landscapes and causes outbreaks.

## Data Availability

The original contributions presented in the study are included in the article and Supplementary material. Further inquiries can be directed to the corresponding author.

## Footnotes

1
https://data.humdata.org/dataset/cod-ab-lka

2
https://hub.worldpop.org

3
http://geodacenter.github.io/

4
http://qgis.org/

## Supplementary material

SUPPLEMENTARY FIGURE S1 Comparison of rate calculations. Different spatial smoothing techniques including empirical Bayesian (EBS) and spatial empirical Bayesian (SBS) were used to calculate case rates in the 6 year time blocks, 2007–2012 **(a)**, 2013–2018 **(b)**, and 2019–2024 **(c)**. When compared to crude/raw rate (RAW), no major differences were observed. RAW rates were used throughout the manuscript.

SUPPLEMENTARY FIGURE S2 District level annual rates from 2007 to 2024. Choropleth maps showing the increase in annual case rates (case/100,000) observed of the 18-year study period.

SUPPLEMENTARY FIGURE S3 District level annual rates from 2007–2024. **(a)** Box plots (Tukey) showing the district level crude case rates (case/100,000) that correspond with the maps in Supplementary Figure S2. **(b)** Table representing the descriptive statistics of the data presented in the box plots in **(a)**.

SUPPLEMENTARY FIGURE S4 Annual precipitation at the district level from 2007 to 2024. Choropleth maps showing the annual precipitation (mm) observed of the 18-year study period.

SUPPLEMENTARY FIGURE S5 Annual precipitation at the district level from 2007 to 2024. **(a)** Box plots (Tukey) showing the district level annual precipitation that corresponds with the maps in Supplementary Figure S4. **(b)** Table representing the descriptive statistics of the data presented in the box plots in **(a)**.
