# Supplementary figures and images for: Spatiotemporal dynamics and environmental trends of reported human leptospirosis in Sri Lanka, 2007–2024
Source: Front Public Health. 2026 May 18;14:1814638. doi: 10.3389/fpubh.2026.1814638 (PMC13223143; doi:10.3389/fpubh.2026.1814638)

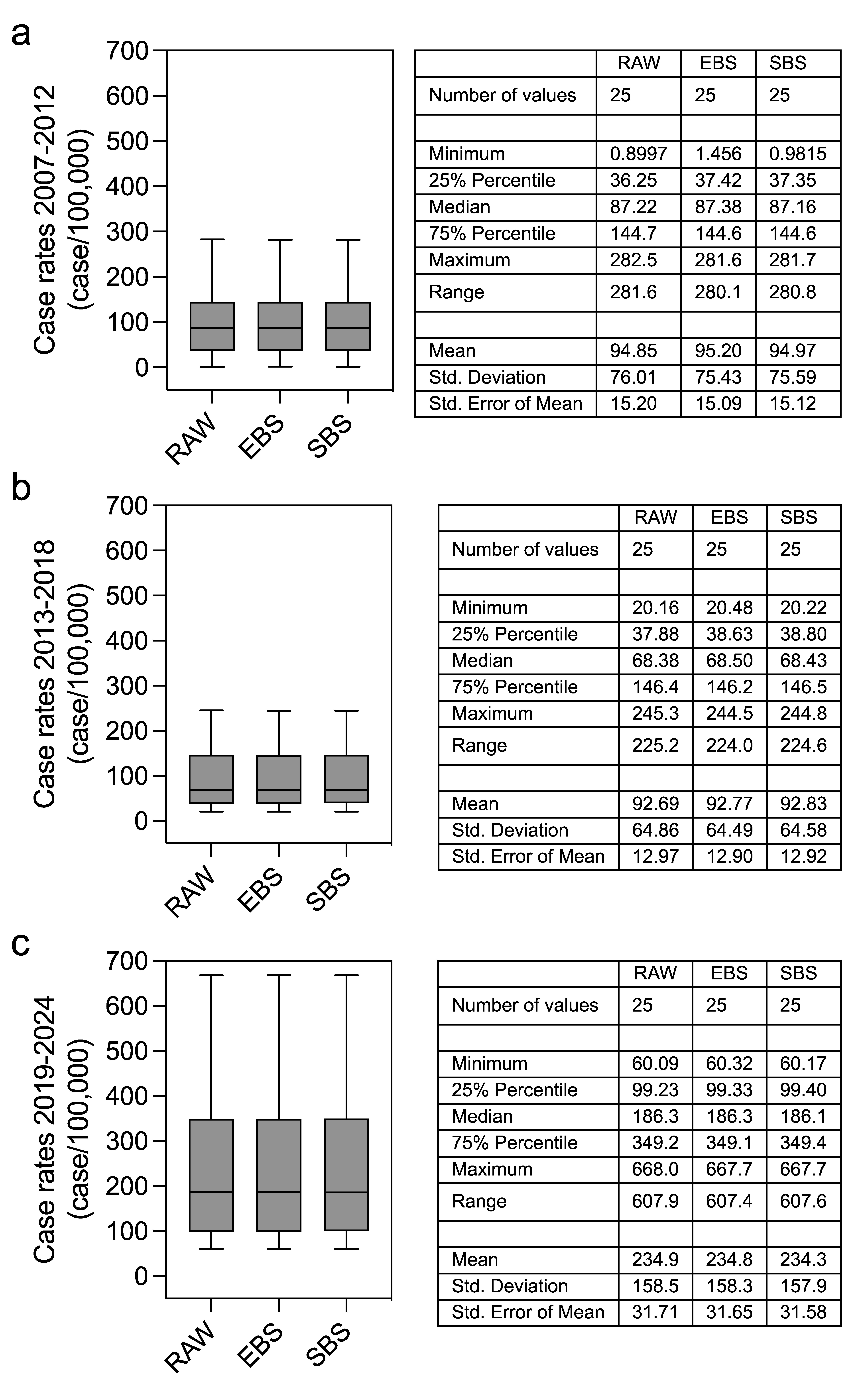

Supplement: SUPPLEMENTARY FIGURE S1 — Comparison of rate calculations. Different spatial smoothing techniques including empirical Bayesian (EBS) and spatial empirical Bayesian (SBS) were used to calculate case rates in the 6 year time blocks, 2007–2012 (a), 2013–2018 (b), and 2019–2024 (c). When compared to crude/raw rate (RAW), no major differences were observed. RAW rates were used throughout the manuscript. [file Image_1.jpeg]

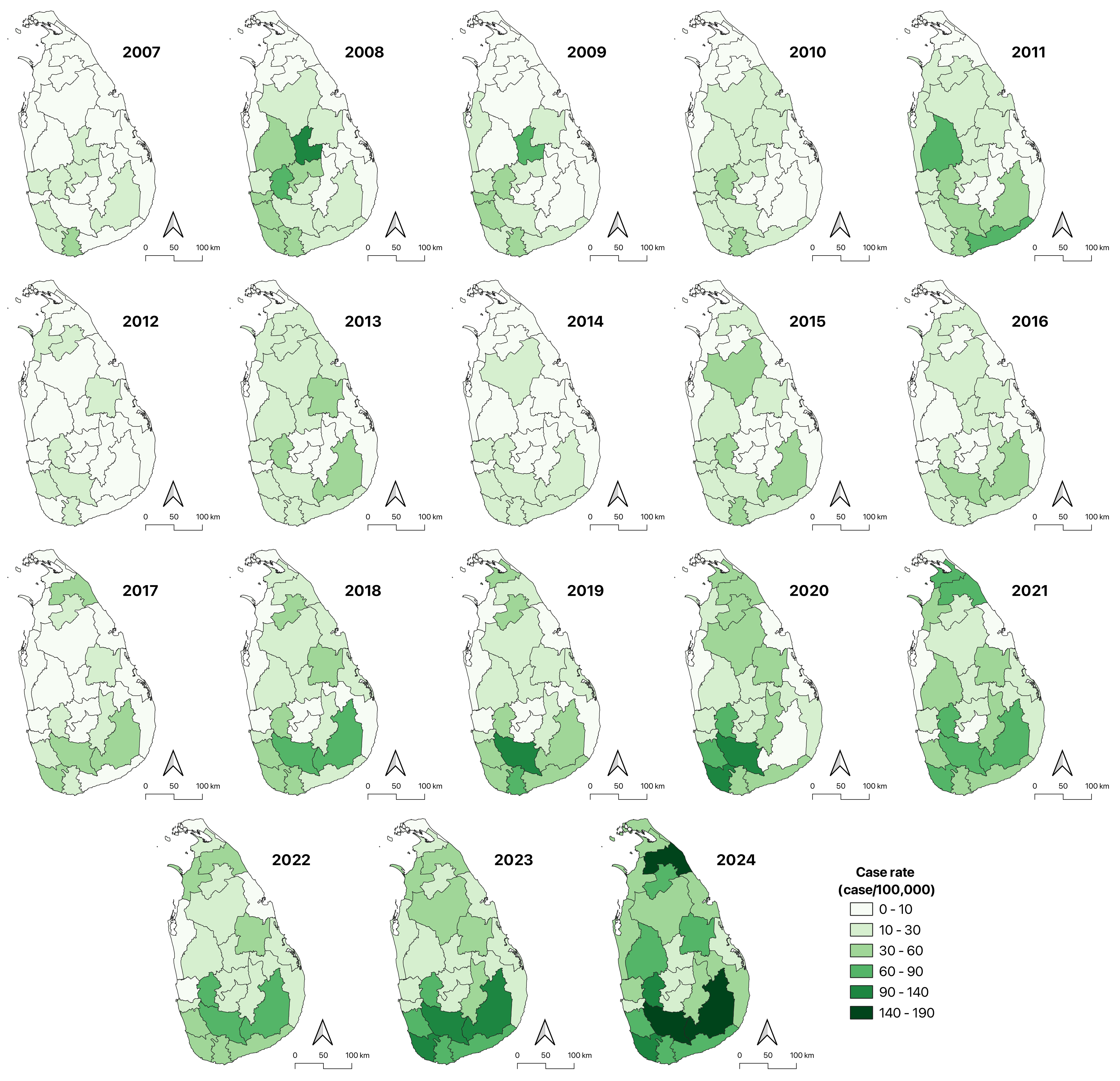

Supplement: SUPPLEMENTARY FIGURE S2 — District level annual rates from 2007 to 2024. Choropleth maps showing the increase in annual case rates (case/100,000) observed of the 18-year study period. [file Image_2.jpeg]

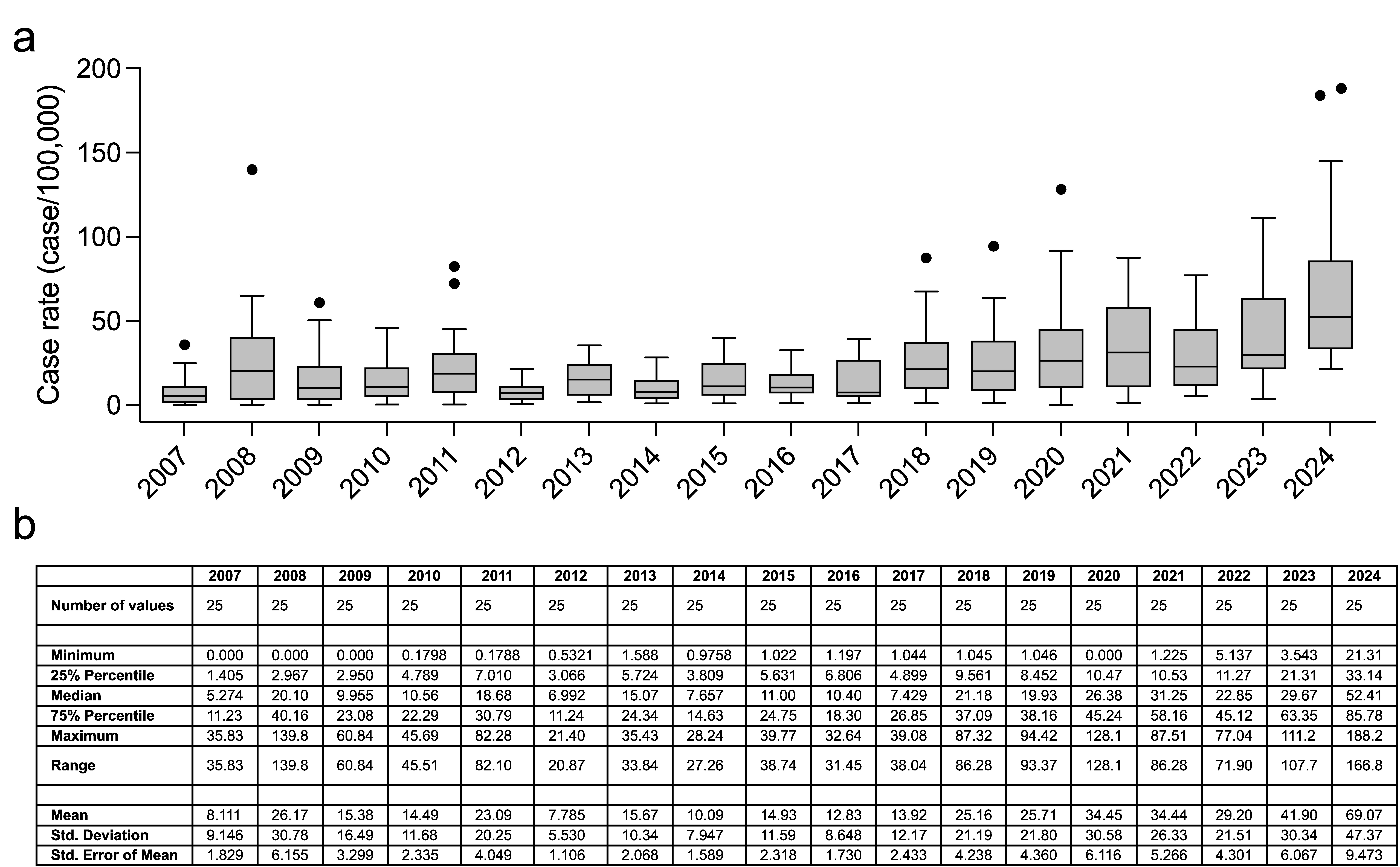

Supplement: SUPPLEMENTARY FIGURE S3 — District level annual rates from 2007–2024. (a) Box plots (Tukey) showing the district level crude case rates (case/100,000) that correspond with the maps in Supplementary Figure S2. (b) Table representing the descriptive statistics of the data presented in the box plots in (a). [file Image_3.jpeg]

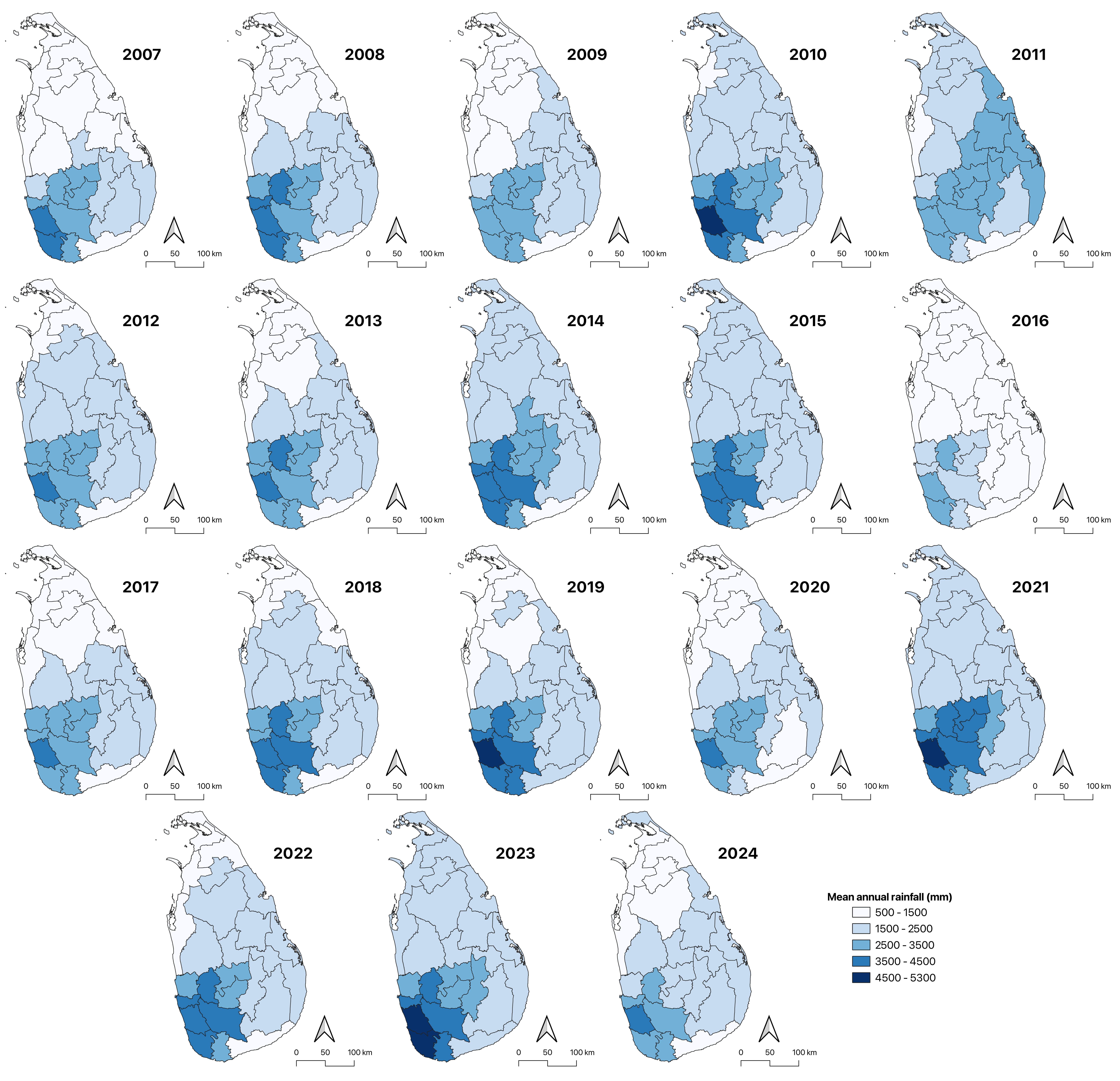

Supplement: SUPPLEMENTARY FIGURE S4 — Annual precipitation at the district level from 2007 to 2024. Choropleth maps showing the annual precipitation (mm) observed of the 18-year study period. [file Image_4.jpeg]

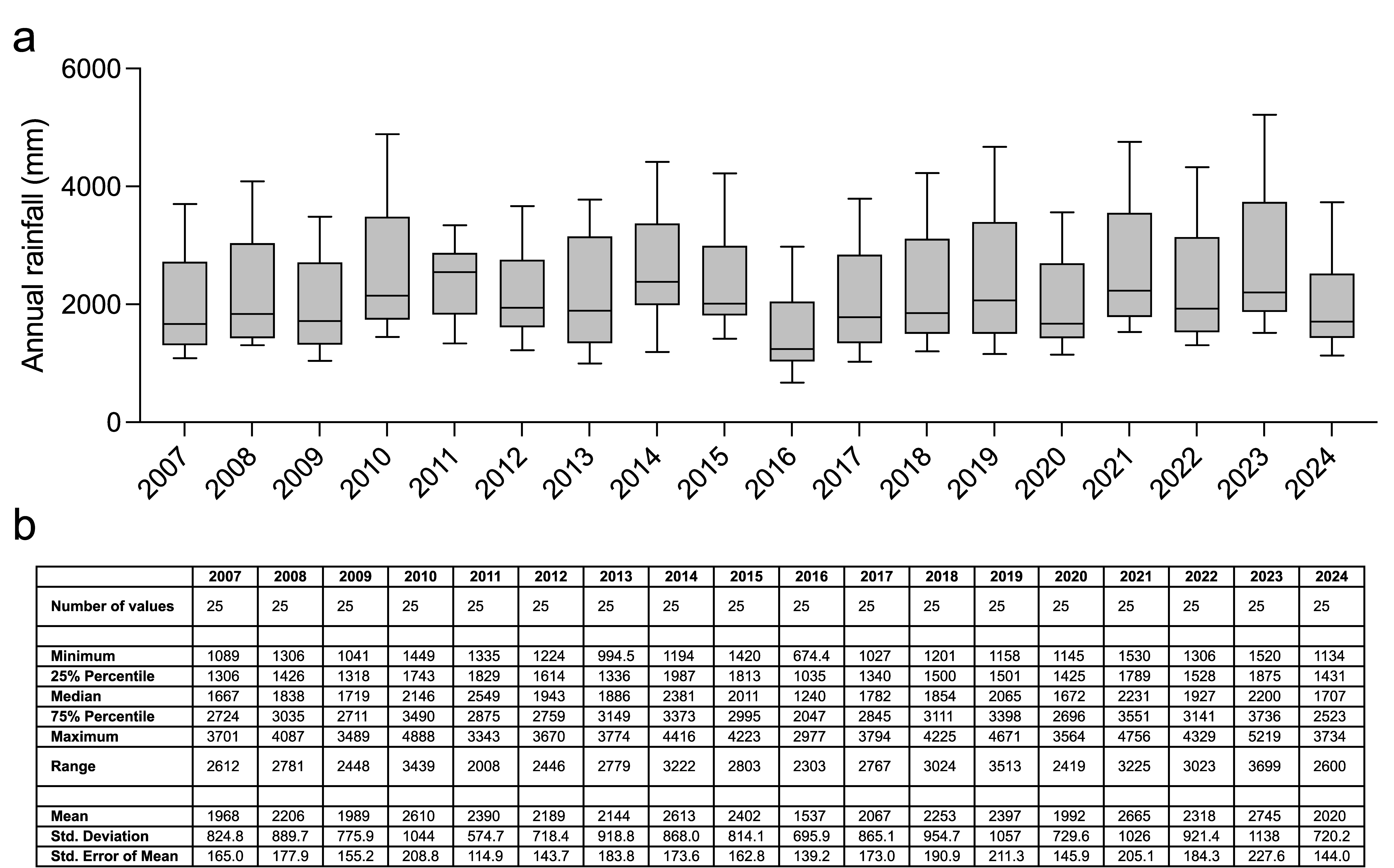

Supplement: SUPPLEMENTARY FIGURE S5 — Annual precipitation at the district level from 2007 to 2024. (a) Box plots (Tukey) showing the district level annual precipitation that corresponds with the maps in Supplementary Figure S4. (b) Table representing the descriptive statistics of the data presented in the box plots in (a). [file Image_5.jpeg]
